# Three New Abietane-Type Diterpenoids from *Callicarpa macrophylla* Vahl.

**DOI:** 10.3390/molecules22050842

**Published:** 2017-05-19

**Authors:** Zhen-Hui Wang, Chao Niu, De-Jun Zhou, Ji-Chuan Kong, Wen-Kui Zhang

**Affiliations:** College of Medicine, Henan Polytechnic University, Jiaozuo 454000, China; rendyx@163.com (C.N.); zhoudj@hpu.edu.cn (D.-J.Z.); kongjichuan@126.com (J.-C.K.); z404105311@me.com (W.-K.Z.)

**Keywords:** *Callicarpa macrophylla* Vahl., abietane-type diterpenoids, NMR, anti-inflammatory activity

## Abstract

Three new abietane-type diterpenoids, named callicapoic acid M3 (**1**), callicapoic acid M4 (**2**) and callicapoic acid M5 (**3**), were isolated from the *Callicarpa macrophylla* Vahl. Their structures were established by spectroscopic techniques (IR, UV, MS, 1D and 2D NMR). All the isolated three compounds were evaluated for inhibitory activity on NO production in LPS-activated RAW 264.7 macrophage cells by using MTT assays. Compounds **1**, **2** and **3** showed potent inhibitory activity, with inhibition rates of 34.47–40.13%.

## 1. Introduction

The genus *Callicarpa* belongs to the family Verbenaceae, with about 190 species widely distributed throughout the tropical and subtropical regions of Asia and Oceanica and parts of America [1,2]. Many *Callicarpa* species are used in Chinese folk medicine for various indications [2]. Compounds that represent a variety of different classes have been reported, including diterpenoids, triterpenoids, flavonoids, phenolic acids, volatile oils and so on [2,3,4,5]. Currently, *Callicarpa macrophylla* Vahl. is used as a folk medicine in China’s Yunnan Province, were the root, the stem, and the leaves are all used in medicine. *Callicarpa macrophylla* Vahl. has a bitter taste, slightly acrid and flat, with clinical actions that eliminate stasis to activate blood circulation and stop bleeding, and it also has detumescence and analgesic actions [6].

**Figure 1:**
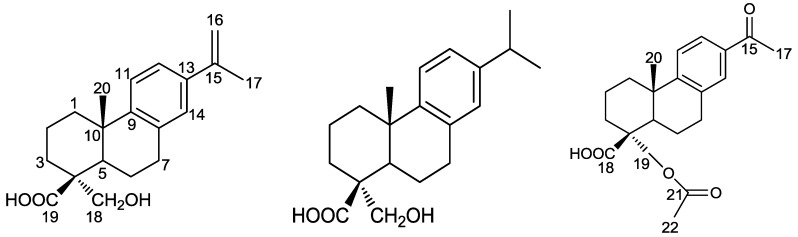
The structures of compounds **1** to **3**.

Past phytochemical studies on *Callicarpa macrophylla* Vahl. Have revealed the presence of pentacyclic triterpenes, sterols [6], and diterpenes [7,8]. Additionally, some of the literature has reported that diterpenoid compounds in *Callicarpa* showed potent anti-inflammatory activities [9,10]. Being interested in finding more biologically active substances from this folk medicine, we further undertook an investigation to explore its phytochemical composition. As a result, three new abietane-type diterpenoids 1–3, named callicapoic acids M 3–5 (Figure 1) were isolated from the dried whole herb of *Callicarpa macrophylla* Vahl. This paper deals with their structural elucidation and anti-inflammatory activity against RAW 264.7 macrophage cells line determined by means of MTT assays.

## 2. Results and Discussion 

Compound **1** (callicapoic acid M3), obtained as white amorphous powder (from acetone), gave the molecular formula C_20_H_26_O_3_, as deduced from the HR-ESI-MS peak at *m*/*z* 315.1958 [M + H]^+^. Its IR spectrum showed hydroxyl (3401 cm^−1^) and carboxyl group (1700 cm^−1^) absorptions, and the UV spectrum showed the presence of an aromatic moiety with maxima at 211 and 250 nm. The ^1^H-NMR spectrum of **1** (Table 1) revealed an isopropenyl group [δ_H_ 2.11 (3H, s), 5.02 (1H, s), 5.32 (1H, s)], one methyl group [δ_H_ 1.16 (3H, s)], a pair of methylene protons [δ_H_ 3.48, 4.16 (each 1H, d, *J* = 5.9 Hz)] bearing an oxygen function, and a 1,2,4 substitution pattern for the aromatic C ring was easily recognized from inspection of other ^1^H-NMR signals [δ_H_ 7.21, 8.25 (each 1H, d, *J* = 8.2 Hz) and 7.12 (1H, s)] and of other C ring aromatized compounds [11,12,13].

The ^13^C-NMR spectrum of **1** (Table 1) confirmed the presence of a benzene ring, in addition to two methyl, six methylene, one methine, one carboxyl, two olefinic carbon signals, and two quaternary carbon signals, as well as an additional methylene carbon (δ_C_ 71.4) attached to an oxygen function. From this information, compound **1** was inferred to be an abietane-type diterpene by comparison with the literature identification data of similar typical abietanes, like the triptobenzenes A–K isolated from *T. wilfordiivar. Regelii* [14] and *T. hypoglaucum* [15].

**Figure 2:**
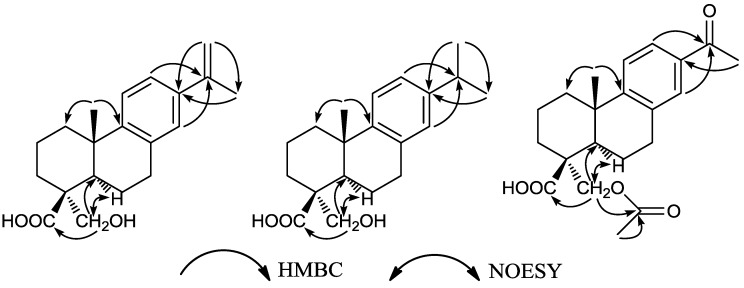
Key HMBC and NOESY correlations for compounds **1** to **3**.

In the HMBC spectrum of **1** (Figure 2), the methyl proton signal (δ_H_ 2.11) of the isopropenyl group correlated with the carbon signals at δ_C_ 138.3 (C-13), 142.9 (C-15), and 111.7 (C-16), and the proton signal δ_H_ 8.25 (H-12) correlated with signals at δ_C_ 142.9 (C-15), 147.2 (C-9), and 126.0 (C-14). In turn, the proton signal δ_H_ 7.12 (H-14) correlated with the signals at δ_C_ 142.9 (C-15), 147.2 (C-9), 123.1 (C-12), and 31.6 (C-7). From these observations, the location of the isopropenyl group at C-13 was inferred. Further, the proton signals at δ_H_ 3.48, 4.16 (H_2_-18) correlated with the signals at δ_C_ 32.0 (C-3), 49.9 (C-4), 47.7 (C-5), and 181.1 (C-19), implied the hydroxyl group in **1** could be assigned to position C-18. In the NOESY spectrum, the proton signals at δ_H_ 3.48, 4.16 (H_2_-18) showed correlations with the proton signal at δ_H_ 1.69 (H-5), and showed no correlation with the methyl proton signals at δ_H_ 1.16 (H_3_-20). Thus, the configuration of the hydroxy methylene at C-4 was confirmed to be α, and the methyl at C-10 was confirmed to be β. The above evidence allowed identification of compound **1** as 18-hydroxy-8,11,13,15-abietatetraen-19-oic acid.

Callicapoic acid M4 (**2**) was isolated as a white amorphous powder (from acetone). Its molecular formula was established as C_20_H_28_O_3_ by the HR-ESI-MS signal at *m*/*z* 339.1927 [M + Na]^+^. The 1D (Table 1) and 2D NMR data of **2** showed the presence of an abietane-type diterpene skeleton, which indicated its structure be similar to that of **1**, except for a side chain isopropyl group, suggested by the appearance of characteristic resonances at δ_H_ 1.21 (3H, s), 1.22 (3H, s), and 2.82 (1H, m); and δ_C_ 24.0, 24.0 and 33.4. In the HMBC spectrum (Figure 2), the methine proton signal (δ_H_ 2.82) of the isopropyl group correlated with C-12 (δ 124.1), C-13 (δ 145.8) and C-14 (δ 126.8). In turn, the proton signals at δ_H_ 6.99 (H-12) and 6.87 (H-14) correlated with the methine signal at δ_C_ 33.4 (C-15). From these observations, the location of the side chain of isopropyl group at C-13 was inferred. The relative configuration of **2** was established by a NOESY experiment, and 4-CH_2_OH, 10-CH_3_ were found to be the same as those of **1**. Accordingly, compound **2** was identified as 18-hydroxy-8,11,13-abietatetraen-19-oic acid (Figure 1) and named callicapoic acid M4 (**2**). Compound **2** is the C-4 epimer of a known compound described in the literature [16].

Callicapoic acid M5 (**3**), a white amorphous powder, possessed a molecular formula of C_21_H_26_O_5_ according to its HR-ESI-MS signal at *m*/*z* 359.1873 [M + H]^+^. The 1D (Table 1) and 2D NMR data of **3** clearly revealed the presence of an abietane-type diterpene skeleton, and also indicated its structure be similar to that of **1** except for the two side chains. The differences between them were that the isopropenyl was replaced by an acetyl at C-13, and the hydroxyl at C-18 was acetylated. The first difference had characteristic resonances at δ_H_ 2.56 (3H, s); and δ_C_ 26.7, 198.3 which implied the presence of an acetyl, while HMBC correlations from protons of methyl at δ_H_ 2.56 to C-13 (δ 134.8) suggested that acetyl was connected to C-13. The second difference in the characteristic resonances at δ_H_ 2.06 (3H, s); and δ_C_ 20.9, 171.0 also implied the presence of an acetyl, HMBC correlations from the methylene protons at δ_H_ 4.10 and 4.49 (H_2_-18) to C-21 (δ 171.0) suggested that the C-18 hydroxyl was acetylated. The relative configuration of 10-CH_3_ was established as β-oriented, 4-CH_2_O- was confirmed to be α-oriented by a NOESY experiment, the same as those of **1** and **2**. Therefore, compound **3** was thus identified as 15-acetyl-19-carbethoxy-8,11,13-abietatetraen-18-oic acid (Figure 1).

Nitric oxide (NO) plays an important role in the inflammatory process [17].The inhibition of NO release may be effective as a therapeutic agent in the inflammatory diseases [18]. Therefore, compounds **1** to **3** were tested for the inhibitory activity against the production of NO in RAW 264.7 stimulated by lipopolysaccharide (LPS). The anti-inflammatory assay was carried out according to the procedure described previously. The results were summarized in Table 2 and indicated that all the three compounds could significantly inhibit NO introduction in LPS-activated RAW 264.7 macrophage cells.

## 3. Experimental

### 3.1. General Procedures

Optical rotations were determined on a 241 MC polarimeter (Perkin-Elmer, Waltham, MA, USA). UV spectra were obtained on Perkin Elmer Lambda 35 UV/VIS Spectrometer. IR spectra were recorded on a IFS 55 spectrophotometer (Bruker, Billerica, MA, USA). The NMR data were recorded on a Bruker AV-600 spectrometer. The HR-ESI-MS data were obtained on a LCT Premier XE time-of-flying mass spectrometer (Waters, Milford, MA, USA). Chromatography was performed on silica gel (200–300 mesh; Qingdao Marine Chemical Group Co. Ltd., Qingdao, China) and ODS (30–50 μm; Tianjin Mical Reagent Co., Tianjin, China). Prep. HPLC was performed on a system comprised of a L-7110 pump and a L-7420 UV spectrophotometric detector set at 203 nm (Hitachi, Tokyo, Japan). A YMC C_18_ reversed-phase column (5 μm, 10 × 250 mm; flow rate 2.0 mL/min) was used.

### 3.2. Plant Material

Dried whole herb of *Callicarpa macrophylla* Vahl. were collected in Yunnan Province, China, in August 2011 and identified by Jincai Lu (School of Traditional Chinese Materia Medica, Shenyang Pharmaceutical University). A voucher specimen (No. 20110801) was deposited in the Research Department of Natural Medicine, Shenyang Pharmaceutical University.

### 3.3. Exaction and Isolation

Dried whole herb of *Callicarpa macrophylla* Vahl. (4.5 kg) was extracted with 50 L 95% EtOH (×3) under reflux conditions for three hours to give a crude extract, which was suspended in H_2_O and successively extracted with petroleum ether (PE), CHCl_3_ and EtOAc to yield a PE-soluble fraction (17.2 g), a CHCl_3_-soluble fraction (261.7 g) and an EtOAc-soluble fraction (55.3 g). A part of the CHCl_3_-soluble fraction (70 g) was subjected to column chromatography (CC, silica gel, gradient of PE-acetone 100:1–0:100) to afford 46 fractions (numbered 1–46). Fractions 30–36 (5.2 g) were separated by CC (ODS, MeCN–H_2_O 30:70, MeCN–H_2_O 40:60) to yield fraction ods40, which was further subjected by semi-preparative reversed-phase HPLC (MeOH/H_2_O with 0.2% HCO_2_H, (62:38, *v*/*v*) as mobile phase, flow rate 3.0 mL/min, wavelength 203 nm), to afford **1** (10.8 mg), **2** (16.4 mg) and **3** (11.2 mg), respectively.

*Callicapoic Acid M3* (**1**). White amorphous powder, [α${]}_{D}^{20}$ +57.0 (MeOH, *c* 0.37). UV (MeOH) λ_max_: 211, 250 nm. IR (KBr) ν_max_ (cm^−1^): 3401, 2930, 1700 and 1561. ^1^H-NMR and ^13^C-NMR spectral data are shown in Table 1. HR-ESI-MS *m*/*z*: 315.1958 (C_20_H_27_O_3_^+^, [M + H]^+^, calc. 315.1960).

*Callicapoic Acid M4* (**2**). White amorphous powder, [α${]}_{D}^{20}$ +22.2 (MeOH, *c* 0.10). UV (MeOH) λ_max_: 205, 266 nm. IR (KBr) ν_max_ (cm^−1^): 3426, 2923, 1630 and 1384. ^1^H-NMR and ^13^C-NMR spectral data are shown in Table 1. HR-ESI-MS *m*/*z*: 339.1927 (C_20_H_28_NaO_3_^+^, [M + Na]^+^, calc. 339.1936).

*Callicapoic acid M5* (**3**). White amorphous powder, [α${]}_{D}^{20}$ +41.2 (MeOH, *c* 0.24). UV (MeOH) λ_max_: 209, 258 nm. IR (KBr) ν_max_ (cm^−1^): 3411, 2927, 1723, 1658 and 1351. ^1^H- and ^13^C-NMR spectral data are shown in Table 1. HR-ESI-MS *m/z*: 359.1873 (C_21_H_27_O_5_^+^, [M + H]^+^, calc. 359.1858).

### 3.4. Anti-Inflammatory Assay

The anti-inflammatory activities of compounds **1** to **3** were evaluated using LPS-induced RAW 264.7 cells. RAW 264.7 macrophages cells (8 × 10^4^ cells/well) were suspended in 100 µL of DMEM supplemented with 10% fetal bovine serum, and precultured in 96-well microplates and 5% CO_2_ in air for 12 h at 37 °C, then test compounds (50 µmol/L) were cultured, and were treated with or without 1 µg/mL LPS for 24 h. NO production in each well was assessed by measuring the accumulation of nitrite in the culture medium using Griess reagent. Cytotoxicity was determined by 3-(4,5-dimethyl-2-thiazolyl)-2,5-diphenyl-2*H*-tetrazolium bromide (MTT) colorimetric assay. Briefly, after 24 h incubation with test compounds, MTT (20 µL, 5 mg/mL in PBS) solution was added to the wells. After 4 h of culturing, the medium was removed and DMSO 100 µL/well was then added to dissolve the formazan produced in the cells. The optical density of the formazan solution was measured with a microplate reader at 490 nm. Z,Z’-6,6’,7,3’α-diligustilide was used as positive control. Each test compound was dissolved in dimethyl sulfoxide (DMSO), and the solution was added to the medium (final DMSO concentration was 0.1%). MTT experiments were repeated three times. The NO inhibitory ratio (%) was calculated by the following formula:

NO inhibitory ratio (%) = (A_570, LPS_ − A_570, sample_)/A_570, LPS_ × 100

## Figures and Tables

**Table 1:** ^1^H- (600 MH_Z_) and ^13^C-NMR (150 MH_Z_) data of compounds **1**–**3** (CDCl_3_).

No.	1		2		3	
	^1^H	^13^C	^1^H	^13^C	^1^H	^13^C
1α	1.37 (m)	38.9	1.38 (t, 12.8)	38.9	1.39 (ddd, 3.5, 9.9, 13.5)	38.6
1β	2.30 (m)		2.28 (d, 12.8)		2.33 (d, 13.5)	
2α	1.70 (m)	19.3	1.69 (d, 11.7)	19.3	1.73 (m)	19.2
2β	2.06 (m)		2.05 (m)		2.12 (m)	
3α	1.13 (m)	32.0	1.12 (m)	32.0	1.19 (dd, 4.6, 13.5)	32.0
3β	2.45 (d, 9.3)		2.45 (d, 11.7)		2.40 (d, 13.5)	
4		49.9		49.9		47.9
5	1.69 (m)	47.7	1.69 (d, 11.9)	47.7	1.78 (d, 12.3)	47.6
6α	2.02 (m)	20.8	2.02 (m)	20.8	2.03 (dd, 5.4, 13.5)	20.8
6β	2.10 (m)		2.08 (m)		2.15 (m)	
7α	2.80 (m)	31.6	2.78 (m)	31.5	2.84 (m)	31.5
7β	2.87 (m)		2.85 (m)		2.97 (dd, 4.7, 16.8)	
8		134.7		134.7		135.4
9		147.2		145.3		153.3
10		38.2		38.1		39.0
11	7.21 (d, 8.2)	125.3	7.17 (d, 8.3)	125.3	7.35 (d, 8.5)	125.9
12	8.25 (d, 8.2)	123.1	6.99 (d, 8.3)	124.1	7.71 (dd, 1.5, 8.5)	126.0
13		138.3		145.8		134.8
14	7.12 (s)	126.0	6.87 (s)	126.8	7.65 (d, 1.5)	129.5
15		142.9	2.82 (m)	33.4		198.3
16*a*	5.02 (s)	111.7	1.21 (s)	24.0		
16*b*	5.32 (s)					
17	2.11 (s)	21.7	1.22 (s)	24.0	2.56 (s)	26.7
18*a*	3.48 (d, 5.9)	71.4	3.48 (d, 9.4)	71.5	4.10 (d, 10.4)	71.6
18*b*	4.16 (d, 5.9)		4.16 (d, 9.4)		4.49 (d, 10.4)	
19		181.1		181.1		180.3
20	1.16 (s)	23.4	1.15 (s)	23.5	1.17 (s)	23.1
21						171.0
22					2.06 (s)	20.9

**Table 2:** Anti-inflammatory effects of compounds **1**–**3** from *Callicarpa macrophylla* Vahl. on LPS-induced RAW264.7 macrophages.

Compound	Conc. (μM)	NO Inhibitory Rate (%)	Cell Viability (%)
**1**	50	37.89 ± 3.28 ^a^	94.26 ± 7.78
**2**	50	34.47 ± 4.35 ^a^	93.58 ± 2.16
**3**	50	40.13 ± 2.45 ^a^	94.76 ± 3.91
Z, Z’-6,6’,7,3’α-Diligustilide ^b^	50	69.37 ± 6.08 ^a^	108.50 ± 1.90

^a^ The three compounds were tested in the same value as 50 μM. ^b^ *p* < 0.01, significantly different from LPS model group. Data were presented as mean ± SD of three independent experiments.
